# Determinants of having no general practitioner in Germany and the influence of a migration background: results of the German health interview and examination survey for adults (DEGS1)

**DOI:** 10.1186/s12913-018-3571-2

**Published:** 2018-10-03

**Authors:** Judith Tillmann, Marie-Therese Puth, Laura Frank, Klaus Weckbecker, Manuela Klaschik, Eva Münster

**Affiliations:** 1 Institute of General Practice and Family Medicine, University of Bonn, 53127 Bonn, Germany; 2 Department of Medical Biometry, Informatics and Epidemiology (IMBIE), University Hospital of Bonn, 53127 Bonn, Germany; 3 Department of Epidemiology and Health Monitoring, Robert Koch Institute, 13353 Berlin, Germany

**Keywords:** Migration, General practitioner, Use of health services, DEGS

## Abstract

**Background:**

It is aspired in the German healthcare system that general practitioners (GPs) act as initial contact for patients and guide through at all steps of medical treatment. This study aims at identifying factors associated with the odds of having no GP within the general population and especially among people with migration background.

**Methods:**

This cross-sectional analysis was based on the “German Health Interview and Examination Survey for Adults” (DEGS1) conducted by the Robert Koch Institute. Descriptive analyses as well as multiple logistic regression models were performed to analyse the impact of a migration background, age, gender, residential area, socioeconomic status (SES) and other factors on having no GP among 7755 participants.

**Results:**

9.5% of the total study population and 14.8% of people with a migration background had no GP, especially men, adults living in big cities and without chronic diseases. The odds of not having a GP were higher for people with a two-sided migration background (aOR: 1.90, 95% CI: 1.42–2.55). Among the population with a migration background, particularly young adults, men, people living in big cities and having a private health insurance showed higher odds to have no GP.

**Conclusions:**

It is necessary to investigate the causes of the differing utilization of healthcare of people with a migration background and, if necessary, to take measures for an equal access to healthcare for all population groups. Further research needs to be done to evaluate how to get young people into contact with a GP.

**Electronic supplementary material:**

The online version of this article (10.1186/s12913-018-3571-2) contains supplementary material, which is available to authorized users.

## Background

Ambulatory medical care in Germany is almost entirely carried out by registered physicians, comprising GPs as well as other specialized physicians [1]. These physicians treat patients, refer them to other specialists or send them to hospitals [1]. Although patients in Germany are generally free to choose their primary healthcare provider, this role should mainly be assumed by general practitioners (GPs). It is aspired that GPs act as guides at all steps of treatment [2]. Better health outcomes through GP-centered healthcare in Germany, especially among older or chronically ill patients, have already been described [3, 4]. International research has been limited to the frequency of use of GPs by the general population [5–8]. Yet it is important to initiate research earlier to find out what drives or deters people to have a GP or not. Especially the establishment of contact by people with a migration background requires particular attention as a less frequent use of healthcare in general [9, 10] and delayed help-seeking behavior have been registered in international studies [11, 12].

The German Federal Statistical Office (Destatis) defines migration background as follows: Either a person his- or herself or at least one parent is born without German nationality [13]. According to the Microcensus 2016, a representative household survey of the official statistics in Germany, 22.5% of the population in Germany shares this characteristic. A further increase in future is predicted [14]. Among this group, Turkey (15.1%), Poland (10.1%) and Russia (6.6%) represent the most common countries of origin [14]. The establishment of contact with primary care by people with a migration background has not yet been analysed in Germany. Only very few findings about the use of healthcare services in general can be summarised: A less frequent utilization of preventative programs, for example medical examinations, preventive dentistry and flu vaccinations as well as rehabilitation measures by citizens with a migration background in Germany have already been described [15–17]. In Danish and Australian studies more visits of emergency departments by patients with a migration background have been reported [18, 19]. Barriers in access to primary care have been identified as the reason in a Danish study [19]. These findings indicate a probable misdistribution of citizens with foreign origin in the healthcare system that needs to be researched in more detail.

The aim of this study was to investigate determinants of not having a GP to visit first in case of any health problem. The focus was set on sociodemographic and health characteristics and their association with having no GP. Furthermore, it was examined whether having no GP differed between people with and without a migration background and which factors were connected to that.

## Methods

The German Health Interview and Examination Survey for Adults (DEGS) is part of the health monitoring carried out by the Robert Koch Institute (RKI) [20]. The RKI is the central federal institution responsible for disease control and prevention. The most current wave (DEGS1) was conducted between November 2008 and December 2011 and included examinations, interviews and tests among 18- to 79-year-olds living in Germany. A random sample from local population registries was combined with the participants of the German National Health Interview and Examination Survey 1998 (GNHIES98), who re-participated. In total, 8152 persons took part, among them 4193 newly invited participants (response 42%) and 3959 who had previously taken part in GNHIES98 (response 62%). The concept and design of DEGS1 have already been described in detail elsewhere [21–23]. The net sample (*n* = 7987) permits representative cross-sectional and time trend analyses. In order to compensate for the empirically lower participation rate of persons without German nationality, an oversampling by a factor of 1.5 was performed. In order to lower language barriers, translations of the consent forms and of the health questionnaires were offered in English, Russian, Serbo-Croatian and Turkish.

In the present analysis, having a GP or not was used as outcome measure and was assessed with the following question: “Do you have a GP to visit first in case of any health problems?”. Only by answering in the affirmative it was assumed that participants had a GP.

The migration background of a participant was considered as potential influence factor on the outcome of having no GP. Within DEGS1, participants with migration background were distinguished between a one-sided and two-sided migration background. People who have immigrated from another country and have at least one parent who was not born in Germany or adults with both parents not born in Germany were regarded as participants with a two-sided migration background. People who were born in Germany and only have one parent who was not born in Germany were considered as participants with a one-sided migration background. Participants who immigrated themselves were considered to be migrants of the first generation. Those with a migration background who were born in Germany were assigned to the second generation. More details have already been published [24].

Further factors included in the analysis were age, gender, residential area, SES, marital status, longer working hours, general state of health, the presence of chronic diseases, type of health insurance and language skills. Knowledge of the German language was only considered for participants with migration background (Table 3). Age, residential area, marital status, general state of health and the presence of chronic diseases were classified as shown in Table 1. The SES was categorised into low, medium or high status depending on the value of a multidimensional index with information on education, professional status and net household income of the participants [23]. The usual number of working hours per week was used to generate a variable (long working hours) with a cut-point at 50 h per week. Health insurance was grouped into statutory health insurance, private health insurance and other (including no insurance, direct payer, foreign health insurance or any other kind of reimbursement). To enable logistic regression analyses among the smaller population group of adults with a migration background, categories of the independent variables have been dichotomised (except “chronic diseases” because of 6% missing values) to reduce the amount of degrees of freedom.

**Table 1:** Characteristics of the study population and percentage with no GP (DEGS1)

	Study population (total) n^a^ (%^b^)	% (95% CI)^b^ of total population with no GP	*p* value^c^
Total	7755 (100)	9.5 (8.4–10.7)	
Migration background			***
One-sided	349 (4.8)	8.0 (5.1–12.2)	
Two-sided	753 (15.1)	16.9 (13.7–20.7)	
No	6552 (80.1)	8.1 (7.1–9.3)	
Gender			***
Male	3682 (49.7)	11.4 (10.0–13.0)	
Female	4073 (50.3)	7.6 (6.4–9.0)	
Age group (years)			***
18–29	1063 (19.1)	17.9 (14.8–21.4)	
30–44	1693 (25.4)	11.8 (9.9–14.1)	
45–64	3051 (36.5)	6.6 (5.5–8.0)	
65–79	1948 (19.0)	3.3 (2.4–4.6)	
Residential area (inhabitants)			***
Rural (< 5000)	1428 (16.2)	5.5 (4.2–7.1)	
Small town (5000 - < 20,000)	1904 (23.3)	7.3 (5.7–9.2)	
Medium-sized town (20,000 - < 100,000)	2244 (29.5)	8.0 (6.6–9.7)	
Big city (100,000+)	2179 (31.0)	14.6 (12.3–17.3)	
Marital status			***
Married	5051 (62.3)	7.4 (6.3–8.6)	
Single	1670 (26.5)	15.9 (13.5–18.6)	
Divorced/widowed	957 (11.2)	6.2 (4.4–8.6)	
SES			***
Low	1167 (18.9)	10.1 (7.9–12.7)	
Medium	4654 (60.6)	7.9 (6.7–9.2)	
High	1903 (20.4)	13.8 (11.4–16.5)	
Excess work (≥50 h/week)			***
Yes	592 (8.3)	13.7 (10.8–17.3)	
No	3839 (54.9)	10.6 (9.1–12.3)	
Non-working/65+ years	3196 (36.8)	6.9 (5.7–8.4)	
General state of health			***
Very good/good	5723 (75.2)	10.9 (9.6–12.4)	
Average/bad/very bad	2005 (24.8)	5.1 (3.8–6.7)	
Chronic diseases			***
Yes	2504 (30.4)	3.7 (2.8–5.0)	
No	4875 (69.6)	11.9 (10.4–13.6)	
Health insurance			***
Statutory	6749 (87.9)	8.3 (7.2–9.6)	
Private	527 (6.7)	19.6 (15.5–24.5)	
Others	468 (5.4)	16.0 (11.9–21.2)	

^a^Unweighted n may not add up to total n due to missing responses

^b^ Weighted results to match the German population structure on 31th December 2010

^c^P values: Comparison between adults having a GP and having no GP, *** *p* < 0.001 ** *p* < 0.01 * *p* < 0.05

Absolute frequencies, percentages and 95% confidence intervals (CI) were determined. Bivariate differences between adults having and not having a GP were evaluated using Chi-square tests and a *p*-value < 0.05 was considered significant. Multiple logistic regression analyses with having no GP as dependent variable were performed. Logistic regression analysis was performed for the total study population, separately for men and women and additionally with focus only on participants with migration background. Adjusted odds ratios (aOR) with 95%-CI were computed. For all independent variables, missing responses were allocated to the reference category in the logistic regression analysis if they did not exceed 5% of cases. Additional analyses restricted to participants with valid data on all independent variables in regression (complete cases) showed similar results to the main analysis (see Additional files 1 and 2). To correct for any deviations of the DEGS1 study population from the German general population, analyses were weighted according to the standardised weighting factor by the Robert-Koch Institute [23]. To take into account both the weighting as well as the correlation of the participants within a community, the confidence intervals were determined with SPSS-25 procedures for complex samples [25].

## Results

The total number of participants aged 18 to 79 years was 7987. Of those, 232 participants were excluded from the analysis due to missing responses regarding data on having a GP. The study population included 7755 participants with a balanced sex ratio and most participants aged between 45 and 64 years; 1102 (19.9%) of them had a migration background (Table 1).

Having no GP was more common in adults with migration background (14.8% in total) than in adults without migration background (8.1%) (Table 3). Men (11.4%) showed significantly higher rates of having no GP than women (7.6%). Adults of the youngest age group (17.9%), adults living in big cities (14.6%) as well as single participants (15.9%) stated significantly more often to have no GP. People with low (10.1%) or high SES (13.8%) stated significantly more often to have no GP than people with a medium SES (7.9%). Having no GP was significantly less likely for adults with an average, bad or very bad general state of health (5.1%), for adults with chronic diseases (3.7%) and for adults with a statutory health insurance (8.3%) (Table 1).

**Fig. 1:**
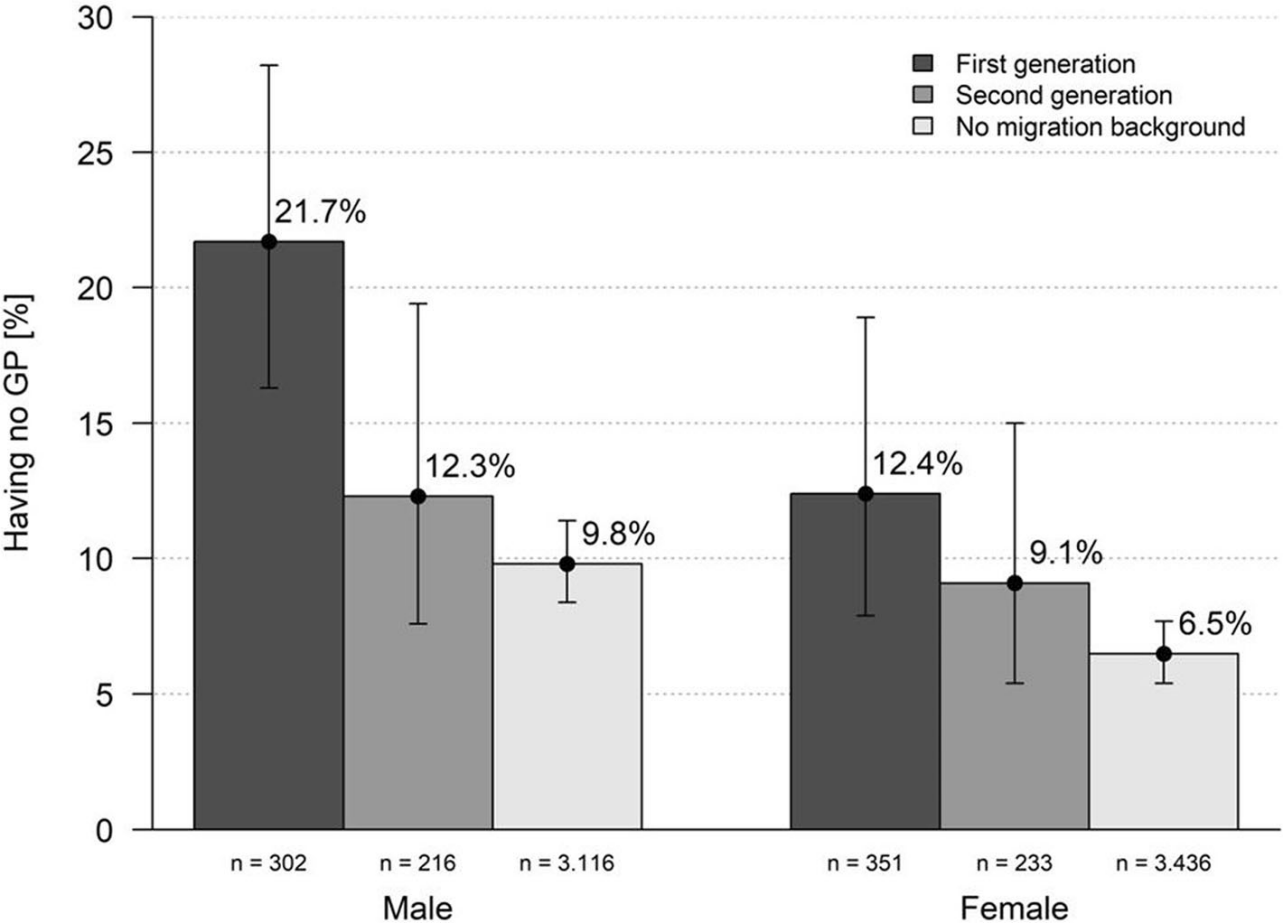
Having no GP (absolute n, weighted percentages with 95%CI) stratified by gender and immigrant generation

People of the first or second generation of migration more frequently had no GP than people without a migration background, especially men (Fig. 1).

The odds of having no GP were higher for adults with a two-sided migration background than for adults without migration background (aOR: 1.90, 95%-CI: 1.42–2.55) (Table 2). Stratification for gender limited that significant effect to male participants with a migration background (aOR: 2.33, 95% CI: 1.54–3.55). Besides, significant effects could be identified in gender, age, residential area, SES, the presence of chronic diseases and the type of health insurance. Men stated more frequently to have no GP than women (aOR: 1.41, 95% CI: 1.15–1.74). Adults of the youngest age group were more than three times as likely at risk compared to adults of the oldest age group (aOR: 3.63, 95% CI: 2.09–6.30), especially women. Men and women living in big cities showed odds of having no GP more than twice as high as men and women living in rural areas (men: aOR: 2.33, 95% CI: 1.53–3.57; women: aOR: 2.76, 95% CI: 1.77–4.31). Women with medium SES had lower odds of having no GP than women with high SES. The presence of chronic diseases reduced the odds of having no GP. Men with private (aOR: 2.45, 95% CI: 1.67–3.61) or any other health insurance (aOR: 2.62, 95% CI: 1.63–4.22) showed more than a two-fold higher odds for having no GP compared to participants with statutory health insurance (Table 2).

**Table 2:** Study population: having no GP with adjusted odds ratios (aOR) and 95% confidence intervals (CI) estimated from logistic regression stratified by gender (DEGS1)

	Having no GP		
	Total (*n* = 7755)	Men (*n* = 3682)	Women (*n* = 4073)
	aOR (95% CI)^a^	aOR (95% CI) ^a^	aOR (95% CI)^a^
Migration background	***	***	
One-sided	0.86 (0.50–1.47)	0.84 (0.41–1.71)	0.94 (0.45–1.99)
Two-sided	1.90 (1.42–2.55)	2.33 (1.54–3.55)	1.43 (0.92–2.22)
No	ref.	ref.	ref.
Gender	**		
Male	1.41 (1.15–1.74)	–	–
Female	ref.	–	–
Age group (years)	***		***
18–29	3.63 (2.09–6.30)	2.63 (1.18–5.88)	5.20 (2.51–10.75)
30–44	2.59 (1.56–4.30)	2.18 (1.06–4.51)	2.95 (1.56–5.57)
45–64	1.78 (1.14–2.76)	1.66 (0.85–3.26)	1.80 (1.01–3.18)
65–79	ref.	ref.	ref.
Residential area (inhabitants)	***	***	***
Big city (100,000+)	2.51 (1.83–3.45)	2.33 (1.53–3.57)	2.76 (1.77–4.31)
Medium-sized town (20,000 - < 100,000)	1.31 (0.94–1.83)	1.12 (0.69–1.82)	1.65 (1.07–2.52)
Small town (5000 - < 20,000)	1.35 (0.95–1.91)	1.29 (0.83–2.01)	1.41 (0.83–2.42)
Rural (< 5000)	ref.	ref.	ref.
Marital status			
Single	1.38 (1.01–1.90)	1.63 (1.02–2.61)	1.09 (0.69–1.74)
Divorced/widowed	1.13 (0.75–1.70)	1.49 (0.83–2.70)	0.86 (0.50–1.48)
Married	ref.	ref.	ref.
SES	*		***
Low	0.89 (0.62–1.28)	0.97 (0.59–1.61)	0.78 (0.49–1.27)
Medium	0.68 (0.51–0.91)	0.90 (0.62–1.29)	0.47 (0.33–0.67)
High	ref.	ref.	ref.
Excess work (≥50 h/week)			
Yes	1.21 (0.88–1.67)	1.18 (0.81–1.74)	1.50 (0.70–3.22)
Non-working/65+ years	1.13 (0.86–1.49)	1.03 (0.65–1.62)	1.24 (0.85–1.79)
No	ref.	ref.	ref.
General state of health			
Average/bad/very bad	0.81 (0.56–1.16)	0.70 (0.43–1.13)	0.96 (0.56–1.63)
Very good/good	ref.	ref.	ref.
Chronic diseases	***	**	**
Yes	0.44 (0.31–0.63)	0.45 (0.28–0.71)	0.45 (0.26–0.80)
No	ref.	ref.	ref.
Health insurance	***	***	
Private	2.23 (1.60–3.12)	2.45 (1.67–3.61)	1.81 (0.96–3.39)
Others	2.23 (1.51–3.31)	2.62 (1.63–4.22)	1.61 (0.89–2.91)
Statutory	ref.	ref.	ref.

^a^Adjusted odds ratios estimated from logistic regression, missing responses were allocated to the reference category (n = 7755). *P* values: *** *p* < 0.001 ** *p* < 0.01 * *p* < 0.05

Characteristics of the population with a migration background (*n* = 1102) are summarised in Table 3. Two thirds were migrants of the first generation living in Germany. The larger part of the population (76.0%) had a two-sided background. 54.3% had another language than German as mother tongue but 85.9% of the participants were native speakers or rated their knowledge of the German language as very good or good. In general, participants with a migration background were younger than people without a migration background and more frequently living in big cities. The amount of people with a low SES was much higher among participants with a migration background (29.5%).

**Table 3:** Comparison of study population with and without a migration background and having no GP with adjusted odds ratios (aOR) and 95% confidence intervals (CI) estimated from logistic regression (DEGS1)

	study population (no migration background) *n* = 6552	study population (migration background) n = 1102	Having no GP (migration population only) n = 1102
	n^a^ (%)	n^a^ (%^b^)	aOR (95% CI)^c^
Having a GP			
Yes	6081 (91.9)	970 (85.2)	–
No	471 (8.1)	132 (14.8)	–
Migration background			
two-sided	–	753 (76.0)	2.02 (0.82–4.97)
one-sided	–	349 (24.0)	ref.
Migration generation			
First generation	–	653 (66.3)	1.01 (0.45–2.26)
Second generation	–	449 (33.7)	ref.
Knowledge of the German language (subjectively)			
Average/bad/very bad	–	111 (14.1)	1.64 (0.92–2.92)
Native speaker/very good/good	–	981 (85.9)	ref.
Gender			*
Male	3116 (50.0)	518 (49.1)	1.78 (1.11–2.85)
Female	3436 (50.0)	584 (50.9)	ref.
Age groups (years)			*
18–44	2220 (41.2)	509 (58.0)	1.67 (1.03–2.71)
45–79	4332 (58.8)	593 (42.0)	ref.
Residential area (inhabitants)			
Big city (100,000+)	1721 (28.2)	425 (42.0)	1.54 (1.01–2.37)
Rural/Small town/Medium-sized town (< 100,000)	4831 (71.8)	677 (58.0)	ref.
Marital status			
Single/divorced/widowed	2237 (38.0)	369 (36.7)	1.06 (0.62–1.83)
Married	4276 (62.0)	724 (63.3)	ref.
SES			
Low	886 (16.0)	253 (29.5)	0.79 (0.39–1.57)
Medium	4004 (62.3)	606 (54.4)	0.57 (0.28–1.15)
High	1656 (21.7)	241 (16.2)	ref.
Excess work (≥50 h/week)			
Yes	516 (8.7)	73 (6.7)	0.92 (0.43–1.97)
No/non-working/65+ years	5946 (91.3)	1012 (93.3)	ref.
General state of health			
Average/bad/very bad	1658 (24.5)	302 (24.9)	0.94 (0.46–1.92)
Very good/good	4872 (75.5)	796 (75.1)	ref.
Chronic diseases			
Yes	2137 (29.9)	322 (23.2)	0.42 (0.17–1.09)
Do not know	303 (5.3)	67 (6.5)	1.53 (0.63–3.73)
No	4112 (64.8)	713 (70.4)	ref.
Health insurance			
Private/others	904 (13.7)	84 (6.0)	2.37 (1.06–5.26)
Statutory	5640 (86.3)	1016 (94.0)	ref.

^a^Unweighted n may not add up to total n due to missing responses

^b^Weighted results to match the German population structure on 31th December 2010

^c^Adjusted odds ratios estimated from logistic regression, missing responses were allocated to the reference category (*n* = 1102). *P* values: *** *p* < 0.001 ** *p* < 0.01 * *p* < 0.05

Logistic regression analyses including only people with a migration background showed significant associations between gender, age, residential area and type of health insurance and having no GP (Table 3). Male participants were again more likely to have no GP (aOR: 1.78, 95% CI: 1.11–2.85) as well as young people (18–44 years) (aOR: 1.67, 95% CI: 1.03–2.71), people with a private or other health insurance (aOR: 2.37, 95% CI: 1.06–5.26) and living in big cities (aOR:1.54, 95% CI: 1.01–2.37). In a model considering only gender, age, residential area, type of health insurance and subjective knowledge of the German language, all variables showed significant influences on having no GP. People without good German language skills had significant higher odds to have no GP in this model.

## Discussion

As it is aspired in German healthcare that GPs are the first point of contact for people with health complaints and guide through at all steps of treatment, this study examines influencing factors on not having a GP for the first time in Germany. Especially people with a two-sided migration background, young adults, men and people living in big cities showed significant higher odds of having no GP.

A special focus has to be set on the result that people with migration background had odds of 1.61 to have no GP compared to people without a migration background. There are several possible barriers this population group may be confronted with in order to find a GP: Migrants of the first generation have to adapt to a new and often different healthcare system. Especially the importance and function of the GP differs a lot depending on the country, e.g. in Turkey there was no family medicine-centered primary care till 2005 and it still differs from the German system [26]. In an Austrian study, a country with a healthcare system quite similar to the German one, the migration status of participants has also been identified as a predictor for consulting specialists without having seen a GP before. Especially men born in Turkey, also representing the most common migration background in Germany, used outpatient departments (OR = 3.05) or hospitals (OR = 5.00) instead of GP services [27]. It is necessary to investigate the causes and backgrounds of the differing utilization patterns of the population with a migration background for example if there is an information deficit about the healthcare system or if there are culturally manifested beliefs about healthcare use.

54.3% of participants with a migration background did not speak German as first language and especially the communication about medical symptoms and terminology might be complicated in another language [28]. In Germany the costs for professional interpreters are not reimbursed in GP practices and have to be paid by the patient [29]. That is why the use of non-professional interpreters like family members or friends is widespread but can cause problems: Shame to talk in front of trusted persons and therefore concealing health problems as well as wrong translations [30]. Since previous studies have already demonstrated the benefits of using professional interpreters in healthcare, it is necessary to build a pool of professional interpreters and to make it possible to bring their services to account in GP practices [31–35]. Culturally determined barriers could also impair to get in touch with physicians: Prejudices and tabooing as well as shame to talk about symptoms and diseases (especially mental diseases) as well as different levels of acceptance of care and therapy forms are widespread [11, 12, 36, 37]. It has to be researched if the use of other medical disciplines is also lower among people with a migration background, reflecting either a general barrier to healthcare or just a lower need, or if there is only a barrier to GPs which would suggest that there is a lack of information about pathways within the German healthcare system. The “Healthy migrant effect”, describing an on average lower mortality and morbidity of immigrants (despite an on average lower socioeconomic status), can also be considered as a possible explanation for the lower amount of people with a migration background having a GP [38, 39]. As this effect mainly occurs in the first generation of immigrants and decreases over time [40] and our results showed no significant difference between migration generations, this effect will not have a large impact.

Differences in having no GP with respect to gender were also in line with previous findings and may be explained by a higher health awareness of women [41, 42]. Differences in the outcome depending on age may result from an insufficient transition process from a pediatrician to a GP and therefore more young people without a GP. Older adults may be more familiar with the German health care system and they are used to have a GP as regular point of contact in case of any medical problem. The difference between people living in urban or rural areas may be explained by the fact that medical specialists are rare in rural areas in Germany and people sometimes have no choice but to establish contact to a GP [43, 44]. A medically unjustified preference of patients in big cities to visit specialists instead of GPs would be a misallocation. In contrast to results reported in most of the literature, not only participants with a low SES but also those with a high SES were less likely to have a GP, especially women [5, 6, 45]. Those with a high SES may again prefer to approach medical specialists. For adults with a low SES, the requirement to pay a “practice fee” of ten Euro, which was raised at that time, may have kept them from getting in contact with a GP.

A new aspect uncovered by the analyses is that every fifth privately insured adult did not have a GP compared to only every twelfth person with statutory health insurance. Waiting times for an appointment at a specialist for privately insured patients are significantly shorter than for statutorily insured patients [46]. In line with this, privately insured adults were found to consult specialists instead of GPs more frequently in a previous analysis of DEGS1 [47]. Further research is necessary why this effect is more prevalent among men. This possible misallocation also manifests in the high number of people visiting emergency departments with minor complaints in Germany instead of making use of GP services [48]. It should also be taken into account that there may be participants who, although they have a GP, consult other health professionals first in the event of illness.

### Limitations

DEGS1 provides a representative sample of the German population aged 18 to 79 years. Still, there is a chance that results are biased as all the information was based on self-reported data. As in many other population-based surveys, chronically ill people might be underrepresented [22]. Besides having a GP to visit first in case of any health problems does not mean that a participant actually makes use of the services of a GP. There may also be participants who contact other medical specialists instead of a GP in case of health problems and therefore negotiating the question. It has to be considered that the DEGS1 dataset is not representative concerning the population with migration background. Despite an oversampling of this group and the application of translated questionnaires, people with migration background are underrepresented [23, 24]. Moreover, translated questionnaires have only been provided in a restricted number of other languages. However, according to weighting of the data, the proportion of persons with a migration background was almost the same as in the general population (weighted: 19.9%, microcensus: 19.2%) [14]. For some variables like immigrant generation the DEGS1 dataset is still biased, because people of the first generation are underrepresented. Stratifying for gender among people with migration background was not possible due to the small sample. The results concerning people with a migration background should not be generalised since there is no homogenous group. When comparing the results with international studies, attention should be paid to how migrant groups are defined because there are no uniform definitions of migration terms. In the present study only the immigrant generation and German language skills were considered as a differentiation of the migration background. Indicators such as country of origin, duration and status of stay and religious orientations should be given more attention.

## Conclusions

This study, for the first time in Germany, examined the differences in frequency of having no GP among people with and without a migration background and characteristics that keep people to have a GP. It is necessary to investigate the causes of the differing utilization of healthcare by people with a migration background and, if necessary, to take measures for an equal access to healthcare for all population groups. Besides young citizens, people living in urban areas and privately insured citizens have to be considered in detail. Further analyses are necessary to understand the patterns of health-seeking behaviour.

## Additional files

Additional file 1: Study population: having no GP with adjusted odds ratios (aOR) and 95% confidence intervals (CI) estimated from logistic regression stratified by gender (DEGS1) – complete case analysis (*n* = 7111). Results of the complete case analysis (n = 7111) (DOCX 22 kb)

Additional file 2: Migration population: having no GP with adjusted odds ratios (aOR) and 95% confidence intervals (CI) estimated from logistic regression (DEGS1) – complete case analysis (*n* = 1001). Results of the complete case analysis (n = 1001) (DOCX 18 kb)

## Abbreviations

aOR: Adjusted odds ratio
CI: Confidence interval
DEGS1: German Health Interview and Examination Survey for Adults (first wave)
GP: General practitioner
SES: Socioeconomic status
