# Supplementary material for: Determinants of having no general practitioner in Germany and the influence of a migration background: results of the German health interview and examination survey for adults (DEGS1)
Source: BMC Health Serv Res. 2018 Oct 3;18:755. doi: 10.1186/s12913-018-3571-2 (PMC6171288; doi:10.1186/s12913-018-3571-2)
Supplement: Supplementary file 1 — Study population: having no GP with adjusted odds ratios (aOR) and 95% confidence intervals (CI) estimated from logistic regression stratified by gender (DEGS1) – complete case analysis (n = 7111). Results of the complete case analysis (n = 7111) (DOCX 22 kb) [file 12913_2018_3571_MOESM1_ESM.docx]

**Additional file 1: Study population: having no GP with adjusted odds ratios (aOR) and 95% confidence intervals (CI) estimated from logistic regression stratified by gender (DEGS1) – complete case analysis (n=7111)**

|  | **Having no GP** | | |
| --- | --- | --- | --- |
|  | **Total** | **Men** | **Women** |
|  | **aOR (95% CI)** | **aOR (95% CI)** | **aOR (95% CI)** |
| **Migration background** | *** | *** |  |
| One-sided | 0.92 (0.53-1.60) | 0.84 (0.40-1.78) | 1.12 (0.53-2.37) |
| Two-sided | 1.81 (1.32-2.48) | 2.10 (1.34-3.31) | 1.43 (0.89-2.31) |
| No | ref. | ref. | ref. |
| **Gender** | ** |  |  |
| Male | 1.46 (1.18-1.81) | - | - |
| Female | ref. | - | - |
| **Age group (years)** | *** |  | *** |
| 18-29 | 3.52 (1.97-6.31) | 2.47 (1.08-5.63) | 5.43 (2.48-11.89) |
| 30-44 | 2.44 (1.42-4.21) | 2.02 (0.96-4.25) | 2.86 (1.42-5.78) |
| 45-64 | 1.64 (1.02-2.64) | 1.53 (0.76-3.08) | 1.63 (0.86-3.09) |
| 65-79 | ref. | ref. | ref. |
| **Residential area (inhabitants)** | *** | *** | *** |
| Big city (100,000+) | 2.50 (1.74-3.59) | 2.31 (1.43-3.73) | 2.81 (1.80-4.39) |
| Medium-sized town (20,000 - <100,000) | 1.22 (0.84-1.79) | 1.01 (0.58-1.74) | 1.64 (1.05-2.55) |
| Small town (5,000 - <20,000) | 1.17 (0.79-1.75) | 1.16 (0.71-1.91) | 1.16 (0.65-2.07) |
| Rural (<5,000) | ref. | ref. | ref. |
| **Marital status** |  |  |  |
| Single | 1.40 (1.01-1.94) | 1.61 (1.01-2.58) | 1.10 (0.68-1.80) |
| Divorced/widowed | 1.04 (0.66-1.62) | 1.28 (0.68-2.41) | 0.83 (0.45-1.50) |
| Married | ref. | ref. | ref. |
| **SES** | * |  | *** |
| Low | 0.84 (0.57-1.25) | 0.93 (0.54-1.59) | 0.73 (0.44-1.23) |
| Medium | 0.67 (0.50-0.91) | 0.90 (0.62-1.30) | 0.44 (0.31-0.64) |
| High | ref. | ref. | ref. |
| **Excess work (≥50h/week)** |  |  |  |
| Yes | 1.17 (0.83-1.64) | 1.12 (0.74-1.69) | 1.52 (0.68-3.40) |
| Non-working/65+ years | 0.99 (0.75-1.31) | 0.88 (0.54-1.43) | 1.10 (0.75-1.62) |
| No | ref. | ref. | ref. |
| **General state of health** |  |  |  |
| Average/bad/very bad | 0.82 (0.54-1.24) | 0.74 (0.42-1.30) | 0.92 (0.52-1.66) |
| Very good/good | ref. | ref. | ref. |
| **Chronic diseases** | *** | ** | ** |
| Yes | 0.47 (0.33-0.69) | 0.46 (0.29-0.74) | 0.51 (0.28-0.91) |
| No | ref. | ref. | ref. |
| **Health insurance** | *** | *** |  |
| Private | 2.08 (1.48-2.93) | 2.21 (1.48-3.32) | 1.86 (0.96-3.59) |
| Others | 2.22 (1.50-3.28) | 2.61 (1.61-4.24) | 1.56 (0.83-2.94) |
| Statutory | ref. | ref. | ref. |

P values: *** p <0.001 ** p < 0.01 * p <0.05
