# Supplementary material for: Determinants of having no general practitioner in Germany and the influence of a migration background: results of the German health interview and examination survey for adults (DEGS1)
Source: BMC Health Serv Res. 2018 Oct 3;18:755. doi: 10.1186/s12913-018-3571-2 (PMC6171288; doi:10.1186/s12913-018-3571-2)
Supplement: Supplementary file 2 — Migration population: having no GP with adjusted odds ratios (aOR) and 95% confidence intervals (CI) estimated from logistic regression (DEGS1) – complete case analysis (n = 1001). Results of the complete case analysis (n = 1001) (DOCX 18 kb) [file 12913_2018_3571_MOESM2_ESM.docx]

Additional file 2: Migration population: having no GP with adjusted odds ratios (aOR) and 95% confidence intervals (CI) estimated from logistic regression (DEGS1) – complete case analysis (n=1001)

|  | | **Having no GP (migration population only)** |
| --- | --- | --- |
|  |  | **aOR (95% CI)^c^** |
| **Migration background** |  | |
| two-sided | | 1.78 (0.71-4.50) |
| one-sided | | ref. |
| **Migration generation** |  | |
| First generation | | 1.00 (0.43-2.28) |
| Second generation | | ref. |
| **Knowledge of the German language (subjectively)** | | |
| Average/bad/very bad | | 1.77 (0.94-3.33) |
| Native speaker/very good/good | | ref. |
| **Gender *** | | |
| Male | | 1.71 (1.03-2.84) |
| Female | | ref. |
| **Age groups (years)*** | | |
| 18-44 | | 1.86 (1.12-3.07) |
| 45-79 | | ref. |
| **Residential area (inhabitants)*** | | |
| Big city (100,000+) | | 1.66 (1.04-2.67) |
| Rural/Small town/Medium-sized town   (< 100,000) | | ref. |
| **Marital status** | | |
| Single/divorced/widowed | | 1.13 (0.64-2.00) |
| Married | | ref. |
| **SES** | | |
| Low | | 0.74 (0.36-1.56) |
| Medium | | 0.60 (0.29-1.25) |
| High | | ref. |
| **Excess work (≥50h/week)** | | |
| Yes | | 0.91 (0.41-2.05) |
| No/non-working/65+ years | | ref. |
| **General state of health** | | |
| Average/bad/very bad | | 0.97 (0.44-2.14) |
| Very good/good | | ref. |
| **Chronic diseases** |  | |
| Yes | | 0.45 (0.17-1.20) |
| No | | ref. |
| **Health insurance** | | |
| Private/others | | 2.24 (0.96-5.24) |
| Statutory | | ref. |

P values: *** p <0.001 ** p < 0.01 * p <0.05
